# Targeting autophagy potentiates the anti-tumor effect of PARP inhibitor in pediatric chronic myeloid leukemia

**DOI:** 10.1186/s13568-019-0836-z

**Published:** 2019-07-15

**Authors:** Yuanyuan Liu, Hong Song, Huanqing Song, Xiaoxia Feng, Chuan Zhou, Zhifang Huo

**Affiliations:** Department of Pediatrics, The Second Affiliated Hospital of Zhengzhou University, Zhengzhou, 450014 China

**Keywords:** Pediatric chronic myeloid leukemia, Talazoparib, Cytotoxicity, Autophagy

## Abstract

Due to its potent cytotoxicity in BRCA-mutated tumors, synthetic lethality elicited by poly (ADP-ribose) polymerase (PARP) inhibitor gives renewed enthusiasm to researching and developing anti-cancer therapies. Chronic myeloid leukemia (CML) is a type of cancers that starts in certain blood-forming cells of the bone marrow. Here, we showed that poly (ADP-ribose) polymerase (PARP) inhibitor talazoparib could induce a concentration-dependent cytotoxicity in CML cells derived from pediatric patients. During talazoparib treatment, autophagy was markedly activated, which was confirmed by the accumulation of autophagosomes, decrease of SQSTM1 and up-regulation of LC3-II. Inhibition of autophagy by pharmaceutical inhibitor chloroquine or small-interfering RNA siATG5 significantly increased the cytotoxicity of talazoparib in pediatric CML cells and elicited synergistic anti-tumor effect in patient-derived xenograft model. Our data demonstrated that autophagy played a cyto-protective role in talazoparib-treated pediatric CML and co-treatment with talazoparib and autophagy inhibitor could induce synergetic anti-tumor effect, providing novel insights for pediatric CML treatment.

## Introduction

Poly (ADP-ribose) polymerases (PARP) played critical roles in a variety of DNA repair processes through sensing single-strand breaks and regulating the subsequent base-excision repair (Fenerty et al. 2018; Karzai et al. 2018). Blocking PARP leads to the accumulation of single-strand breaks, which will activate homologous recombination repair (Ashworth 2008; Rouleau et al. 2010). BRCA1/2 are the critical proteins in homologous recombination and the mutation of BRAC1/2 in tumors may lead to defects in DNA repair (Farmer et al. 2005). Since researchers found that BRAC1/2-mutant tumors were sensitive to PARP inhibition a decade ago, several PARP inhibitors olaparib, rucaparib, niraparib and talazoparib have been approved by FDA to treat germline BRCA-mutated advanced ovarian cancer and breast cancer (Sun et al. 2017; Zimmer et al. 2018). However, indications for PARP inhibitors represent only a small fraction of cancers that carrying BRCA mutation. Chronic myeloid leukemia (CML) is a type of leukemia characterized by the abnormal accumulation of myeloid cells and CML in childhood accounts for 10% of all cases (Tang et al. 2018; Ureshino et al. 2018). Here, in this article we investigated the anti-tumor effect of PARP inhibitor talazoparib in pediatric CML and the underlying mechanisms.

Similar to nearly all contemporary therapies for patients with malignance, efficacy decreases with successive lines of treatment (Hartley et al. 2018; Johnson et al. 2011). Autophagy is an evolutionarily conserved cellular process, which plays an important role in tumor microenvironment (Zhang et al. 2018b). Many anti-tumor agents, including asparaginase, arginase, cisplatin and immune checkpoint inhibitor, activated autophagy in cancer cells (Chen et al. 2017; Shen et al. 2017; Zhang et al. 2018a). Blocking autophagy significantly increased the anti-tumor effect of these agents, demonstrating that autophagy could be an efficient target to enhance the efficacy of anti-tumor regents. Increasing studies demonstrated that autophagy was involved in DNA damage repair especially in tumor cells (Karantza-Wadsworth et al. 2007; Liu et al. 2015). Inhibition of autophagy degraded checkpoint kinase 1 and further diminished DNA damage repair. Defective autophagy by allelic loss of beclin 1 increased DNA damage and genomic instability in breast cancer (Karantza-Wadsworth et al. 2007). Herein, we hypothesized that autophagy was triggered in PARP inhibitor-treated pediatric CML cells and combining used of talazoparib and autophagy inhibitor could elicit synergetic anti-tumor effect in pediatric CML.

In this study, first we investigated the cytotoxicity of talazoparib in CML cells derived from pediatric patients. Then autophagy was determined in talazoparib-treated CML cells. Finally, we confirmed the role of autophagy by pharmaceutical inhibitor and small-interfering RNA (siRNA) in vitro and in vivo. Our data showed that talazoparib could reduce the cell viability of CML cells and co-treatment with talazoparib and autophagy could induce synergetic anti-tumor effect, providing novel strategy for pediatric CML patients.

## Materials and methods

### Reagents

Talazoparib and autophagy inhibitor chloroquine were ordered from Selleck (Shanghai, China). CCK-8 kit was purchased from Beyotime Biotechnology (Haimen, China). Hoechst 33342 and Cyto-ID green were obtained from ENZO Life Science (Farmingclale, NY, USA). The primary antibodies anti-SQSTM1, anti-LC3-I/II and anti-β-actin were obtained from Cell Signaling Technology (Danvers, MA, USA). HRP-conjugated goat anti-mouse/rabbit IgG were obtained from MR Biotech (Shanghai, China).

### Cells

Leukemic cells (P#1 and P#2) were purified by Ficoll-Hypaque (Sigma-Aldrich, USA) from peripheral blood of two 12-years-old pediatric CML patients presenting with typical immunophenotype and morphology. CML cells were cultured in RPMI-1640 containing 10% FBS (Gibco, USA) at 37 °C in a humidified atmosphere of 5% CO_2_ incubator.

### Cytotoxicity assay

CCK-8 assay was used to measure cell viability. Briefly, CML (P#1 and P#2) cells were seeded into 96-well plates in a concentration of 5 × 10^4^ cells/mL. After treatment with talazoparib and/or autophagy inhibitor at indicated concentrations, CCK-8 was added to each well for 4 h at 37 °C. Then the optical density value was detected by a UV spectrophotometry at 450 nm.

### Transmission electron microscopy

CML cells were treated with talazoparib for 48 h. Then the samples were harvested and processed under the instructions (Shenoy et al. 2018). A transmission electron microscope (TEM, JEM 1410) was used to detect the sliced samples. Micrographs were obtained at the magnification of 7000× or 20,000×.

### Immunoblot analysis

After treatment with various concentration of talazoparib and/or autophagy inhibitors for 48 h, CML cells were subjected to protein extracted extraction and equivalent amounts of the extraction were separated by SDS-PAGE and transferred onto PVDF membranes. Following blockage of nonspecific sites with 5% bovine saline albumin, the membranes were incubated with primary antibodies and subsequently subjected to secondary antibodies. ImageJ Software was used to quantify the resulting bands.

### Confocal microscopy

CML cells were treated with talazoparib for 48 h and rapamycin was employed as a positive control. Then, cells were incubated with Cyto-ID and Hoechst 33342 following the manufacturer’s instructions (Samaniego et al. 2018). Subsequently, a confocal microscopy (Carl Zeiss LSM710, Carl Zeiss, Germany) was employed to observe the cells.

### RNAi

Human ATG5 siRNA and non-silencing scrambled control (SCR) siRNA were obtained from Sangon Biotech (Shanghai, China). Cells were transfected with 50 nM of siRNA and Lipofectamine™ RNAiMAX Transfection Reagent (Invitrogen, USA) for 24 h. Then the cells were collected and the expression level of ATG5 was detected to the transfection efficiency.

### Patient-derived xenograft (PDX) model

Male BALB/c nude mice (6 weeks) were subcutaneously injected with 1 × 10^7^ of patient-derived cells (P#1) suspended in 50% Martigel Matrix (Corning, USA) to establish CML patient-derived xenograft model (Hu et al. 2018). Treatment was started once the volume of the tumor reached approximately 100 mm^3^ (volume = length × width × width/2). Mice were randomized into four cohorts as indicated. Talazoparib (50 mg/kg) was administrated orally once a day and chloroquine (50 mg/kg) was intraperitoneally injected once a day. Tumor volume was measured once every 3 days.

### Statistical analysis

Results were shown as mean ± SD and comparisons were performed using Student’s t-test. Value of *P* < 0.05 was considered statistically significant.

## Results

### PARP inhibition induced cytotoxicity in CML cells

**Fig. 1:**
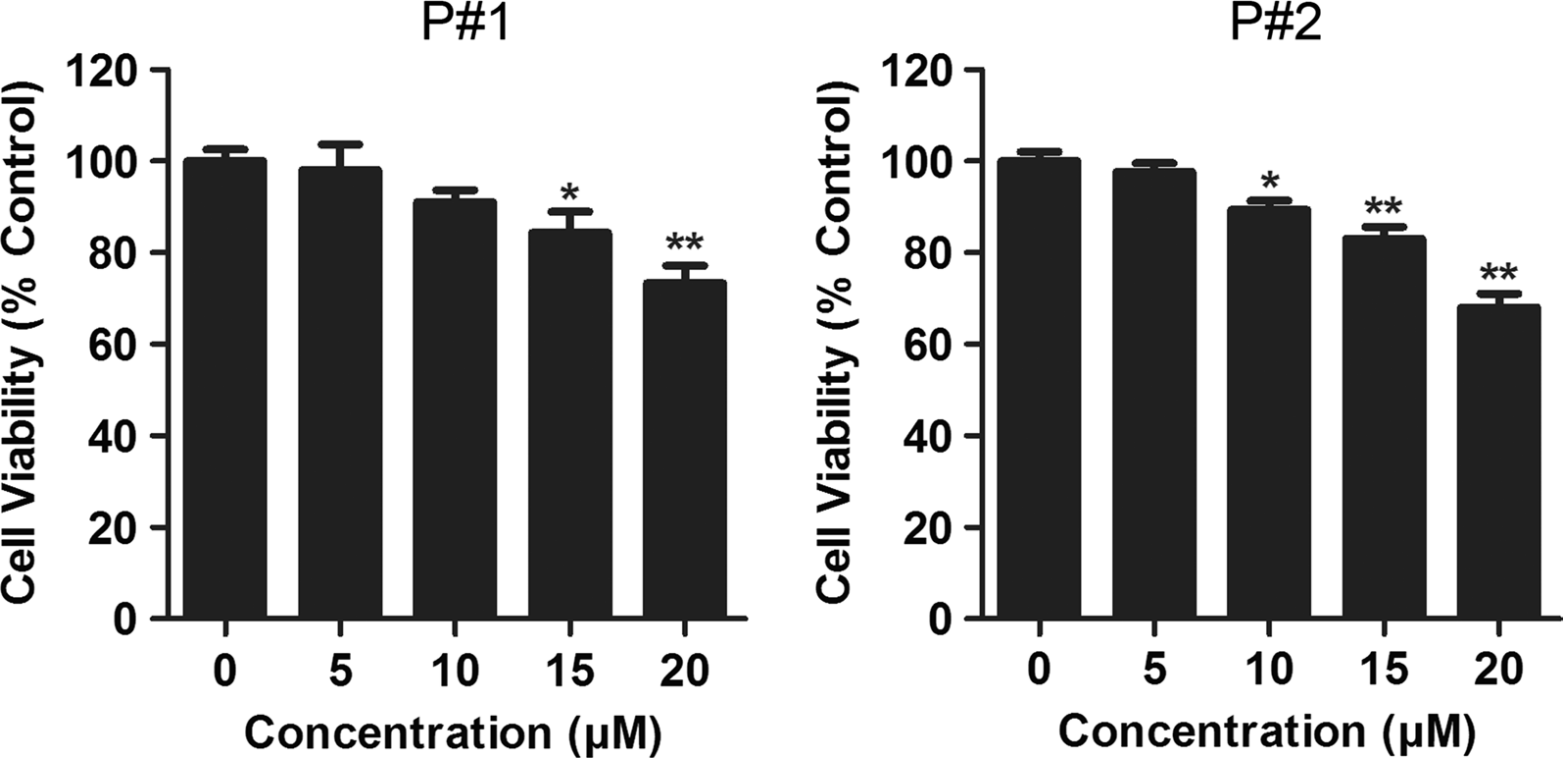
PARP inhibition reduced the cell viability of primary CML cells. CML P#1 and P#2 cells were incubated with indicated concentration of PARP inhibitor talazoparib for 48 h. Cell viability was detected by CCK-8 assay. (*N* = 3, mean ± SD, **P < 0.01)

As PARP is involved in DNA repair and over-expressed in various cancers, inhibitors targeting PARP have gained huge success in clinic (LaFargue et al. 2019). In this study, first we investigated whether inhibition of PARP by talazoparib could induce cytotoxicity in primary CML P#1 and P#2 cells in vitro. The cell viability of CML cells in response to various concentrations of talazoparib was measured by CCK-8 assay. As shown in Fig. 1, treatment with talazoparib for 48 h decreased the cell growth ability of CML in a dose-dependent manner. Moreover, a significant decrease of cell viability was observed in CML cells incubated with 20 μM talazoparib.

### PARP inhibitor talazoparib triggered autophagy in CML cells

**Fig. 2:**
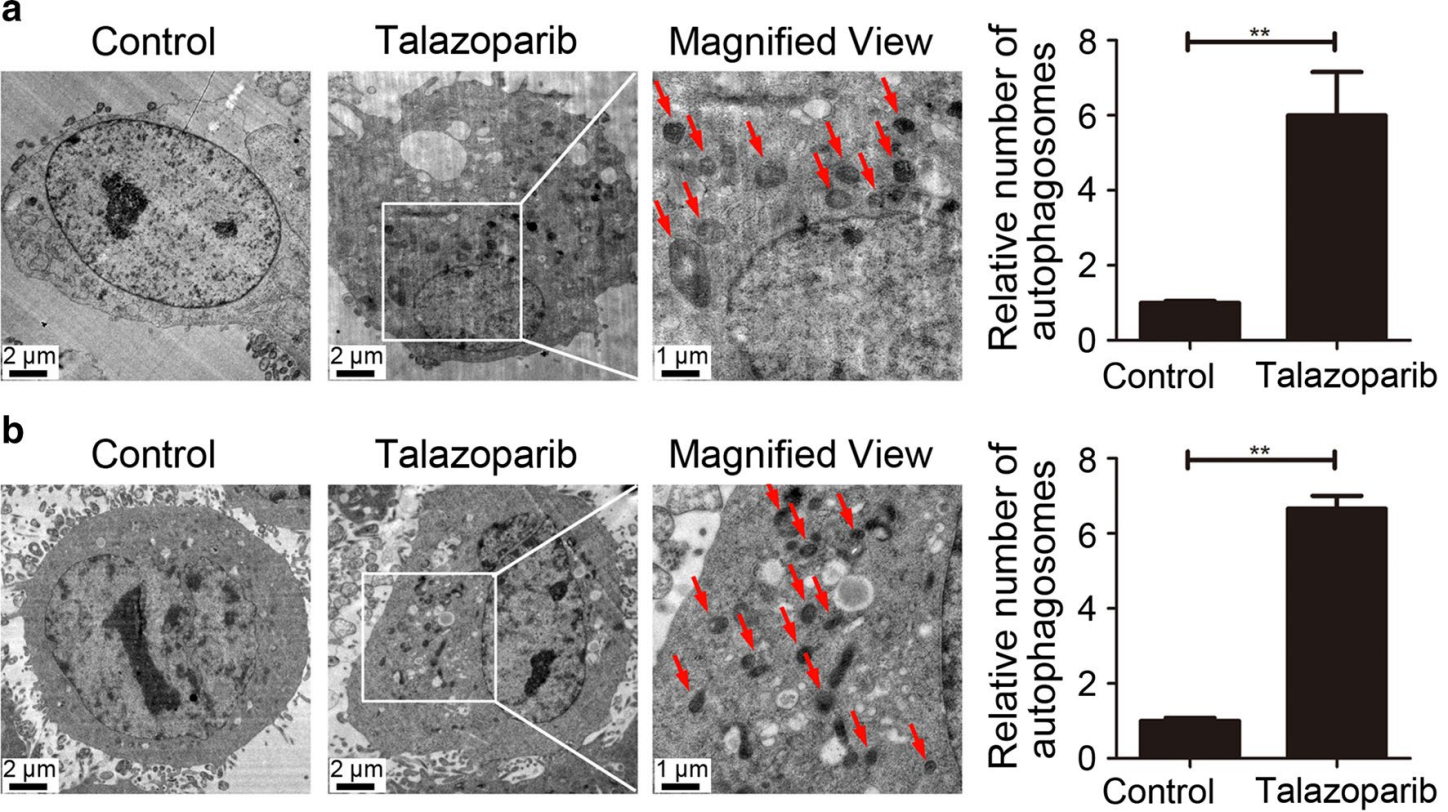
Autophagosomes accumulation was induced by PARP inhibitor talazoparib in CML cells. Ultrastructural analysis of P#1 cells (**a**) and P#2 cells (**b**) treated with 20 μM of talazoparib for 48 h by TEM. The relative number of autophagosomes were quantified by Image J software (*N* = 3, mean ± SD, **P < 0.01)

**Fig. 3:**
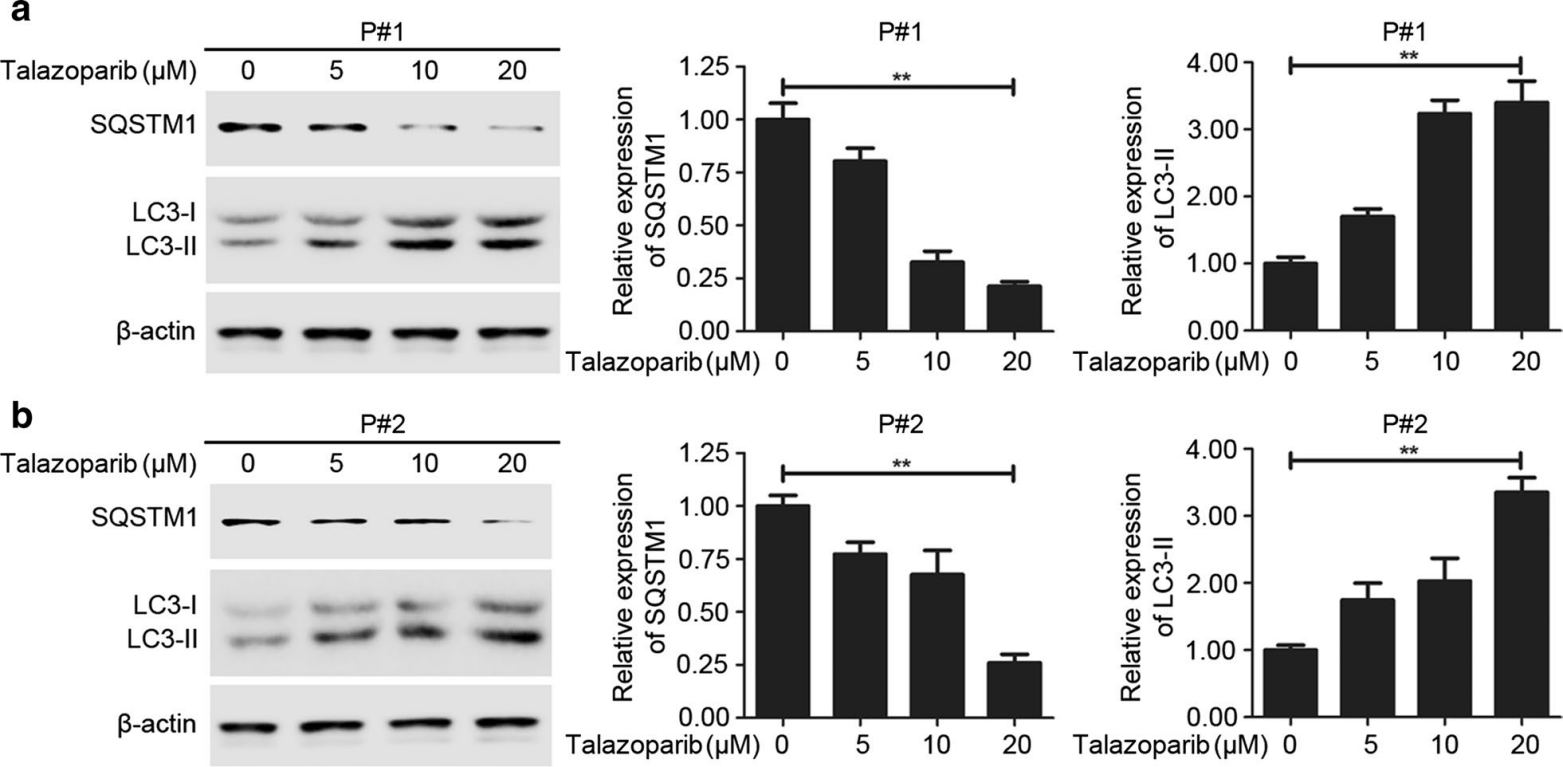
PARP inhibitor talazoparib triggered autophagy initiation in CML cells. Analysis of autophagy markers, SQSTM1 and LC3-I/II in CML P#1 (**a**) and P#2 (**b**) cells after treatment with talazoparib (20 μM) for 48 h. Densitometric values were measured by Image J software and the data was shown as mean ± SD of three dependent experiments (**P < 0.01)

**Fig. 4:**
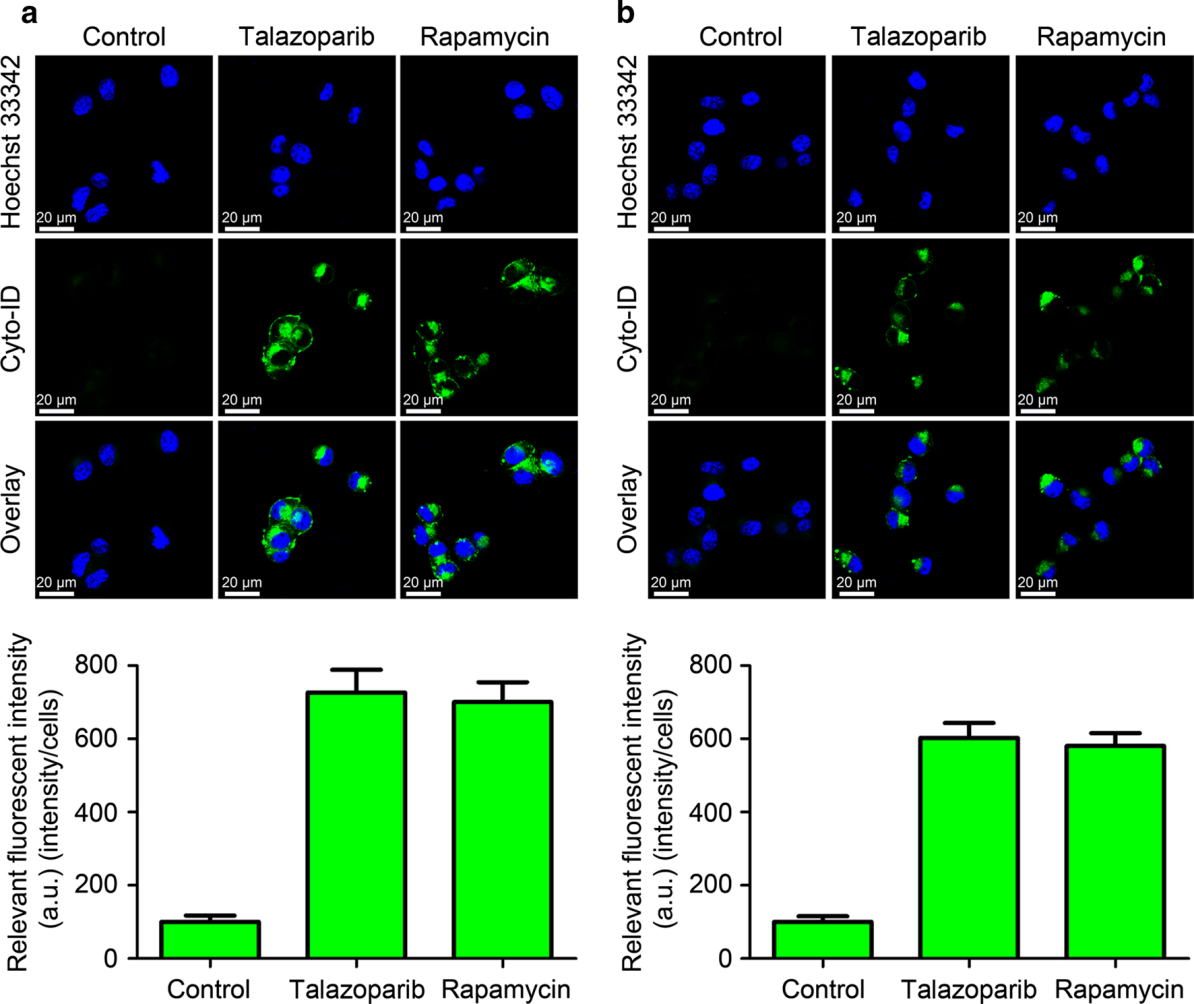
PARP inhibitor talazoparib induced autophagosomes accumulation in CML cells. Cyto-ID green dye was employed to detect the accumulation of autophagosomes in CML P#1 (**a**) and P#2 cells (**b**) after exposure to talazoparib (20 μM) for 48 h. Rapamycin was used as the positive control (*N* = 3, mean ± SD)

Increasing literatures showed that autophagy was involved and played critical role in cancer therapy (Zhang et al. 2017). Hence, we tried to investigate whether autophagy was involved in CML cells treated with talazoparib. First, ultrastructural analysis by TEM was applied to determine the hallmark of autophagy, autophagosomes, which was characterized by double-membraned vesicles. After talazoparib administration for 48 h, CML P#1 cells showed a significant accumulation of autophagosomes in the cytoplasm (Fig. 2a). Similar result was also observed in the cytoplasm of CML P#2 cells (Fig. 2b). These data demonstrated that autophagy was initiated in human CML cells after talazoparib treatment. Then, immunoblot analysis was used to detect the level of two autophagy-related protein, sequestosome 1 (SQSTM1) and microtubule-associated protein 1 light chain3 (LC3). Figure 3 showed that talazoparib obviously increased the level of LC3-II and reduced the level of SQSTM1 in CML cells in a concentration-dependent manner after talazoparib treatment. Furthermore, Cyto-ID, an autophagic vesicle-specific dye was employed to confirm talazoparib-induced autophagy in CML cells. Similar to the positive control rapamycin-treated cells, cells exposure to talazoparib increased the punctate fluorescence in the cytoplasm. In summary, these results showed that PARP inhibitor talazoparib activated autophagy in CML cells (Fig. 4).

### Autophagy inhibition potentiated the cytotoxicity of talazoparib in CML cells

**Fig. 5:**
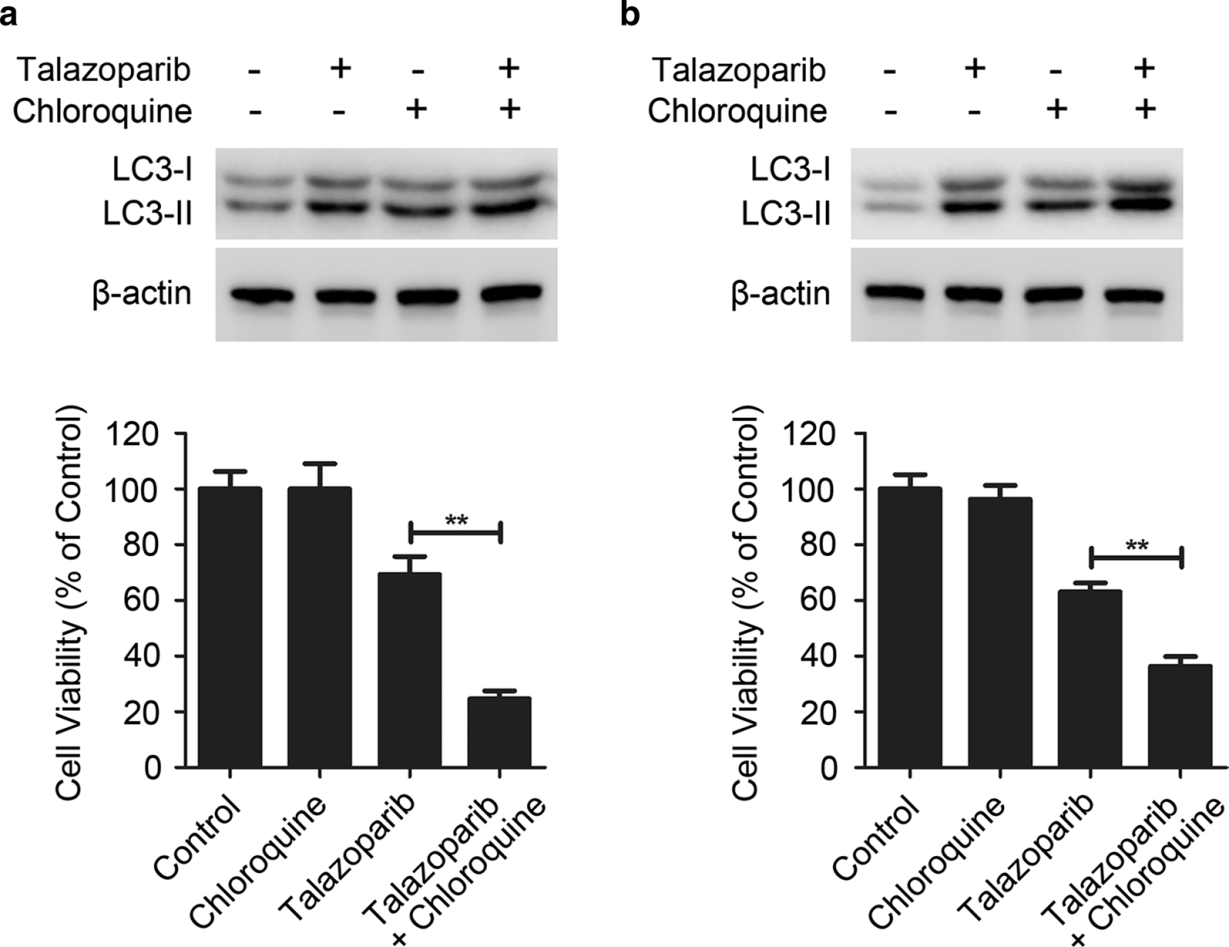
Autophagy inhibition potentiated the cytotoxicity of talazoparib in CML cells. CML P#1 cells (**a**) and P#2 (**b**) were treated with 20 μM of talazoparib in with/without of 20 μM of chloroquine. The expression of LC3-I/II was detected by immunoblot analysis. Cell viability was measured by CCK-8 assay (*N* = 3, mean ± SD, **P < 0.01)

**Fig. 6:**
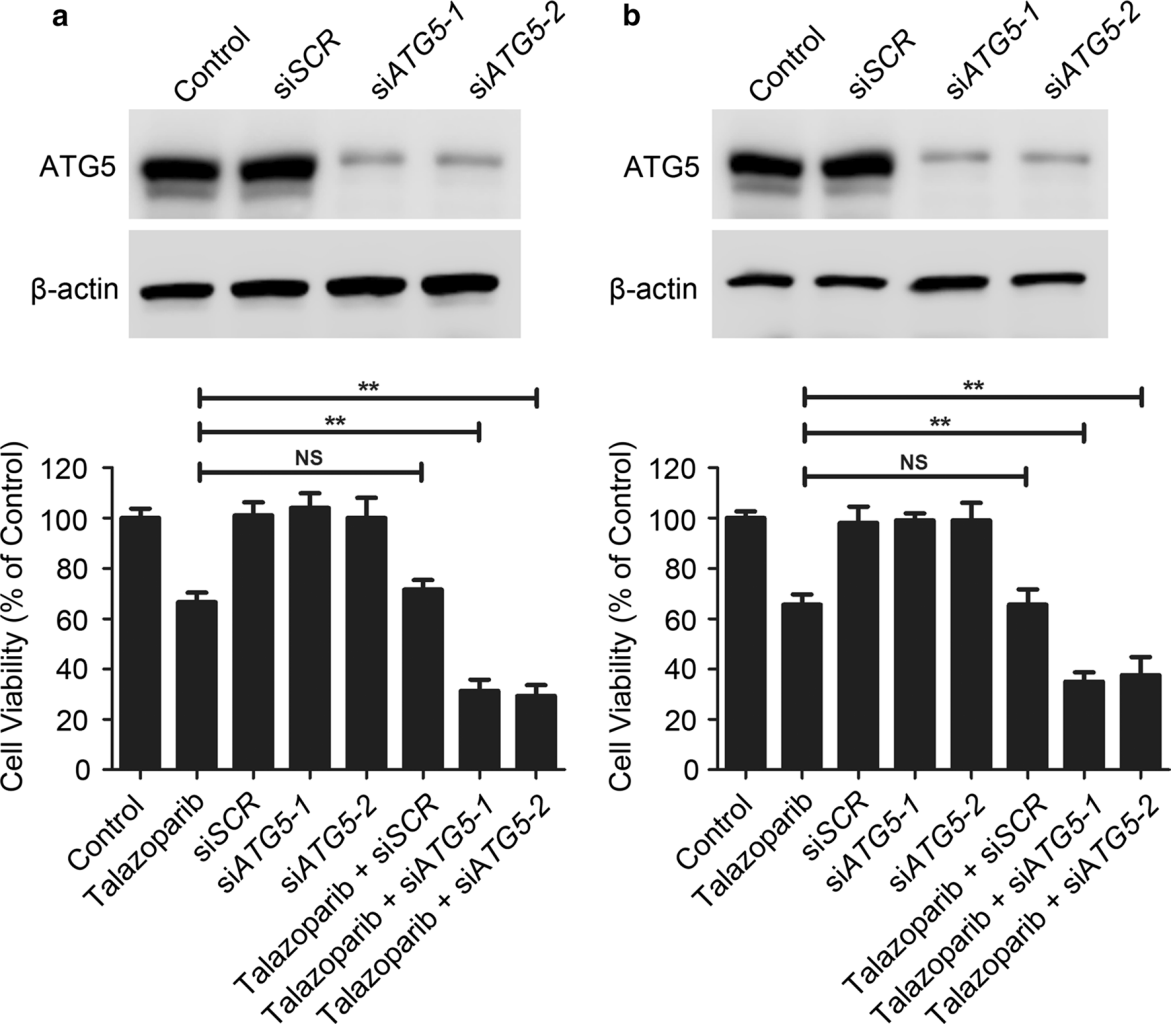
Knockdown of ATG5 enhanced the cytotoxicity of talazoparib in CML cells. P#1 (**a**) and P#2 (**b**) were transfected with ATG5 siRNA for 48 h, and immunoblot analysis was used to determine the protein level of ATG5. Cell viability was measured by CCK-8 assay (*N* = 3, mean ± SD; *NS* no significance; **P < 0.01)

To investigate the effect of autophagy in talazoparib-induced cytotoxicity in CML cells, chloroquine, a classic autophagy inhibitor was used to block talazoparib-triggered autophagy. Immunoblot analysis showed that the level of LC3-II in the cells treated with talazoparib and chloroquine was obviously increased than that in talazoparib-treated cells, indicating that talazoparib-induced autophagy was successfully blocked by chloroquine (Fig. 5). Chloroquine did not induced significant cytotoxicity in CML P#1 and P#2 cells. Meanwhile, talazoparib in combination with chloroquine significantly potentiated talazoparib-triggered cytotoxicity in CML cells (Fig. 5). To further confirm the cytoprotective role of autophagy in talazoparib-induced cytotoxicity, ATG5, a core autophagy molecular in the formation of autophagy initiation, was knocked down. Immunoblot analysis showed that siRNA-*ATG5* selectively decreased the protein level of ATG5 in CML P#1 and P#2 cells when compared with the non-target control (Fig. 6). After knockdown of ATG5 in talazoparib-treated CML cells, talazoparib-induced cytotoxicity was significantly enhanced (Fig. 6). Therefore, our results demonstrated that talazoparib-induced autophagy played a cytoprotective role in CML cells. Autophagy inhibition could significantly potentiate the anti-tumor effect of PARP inhibitor talazoparib in CML.

### Talazoparib combined with autophagy inhibitor induced synergetic anti-tumor effect in CML PDX model

**Fig. 7:**
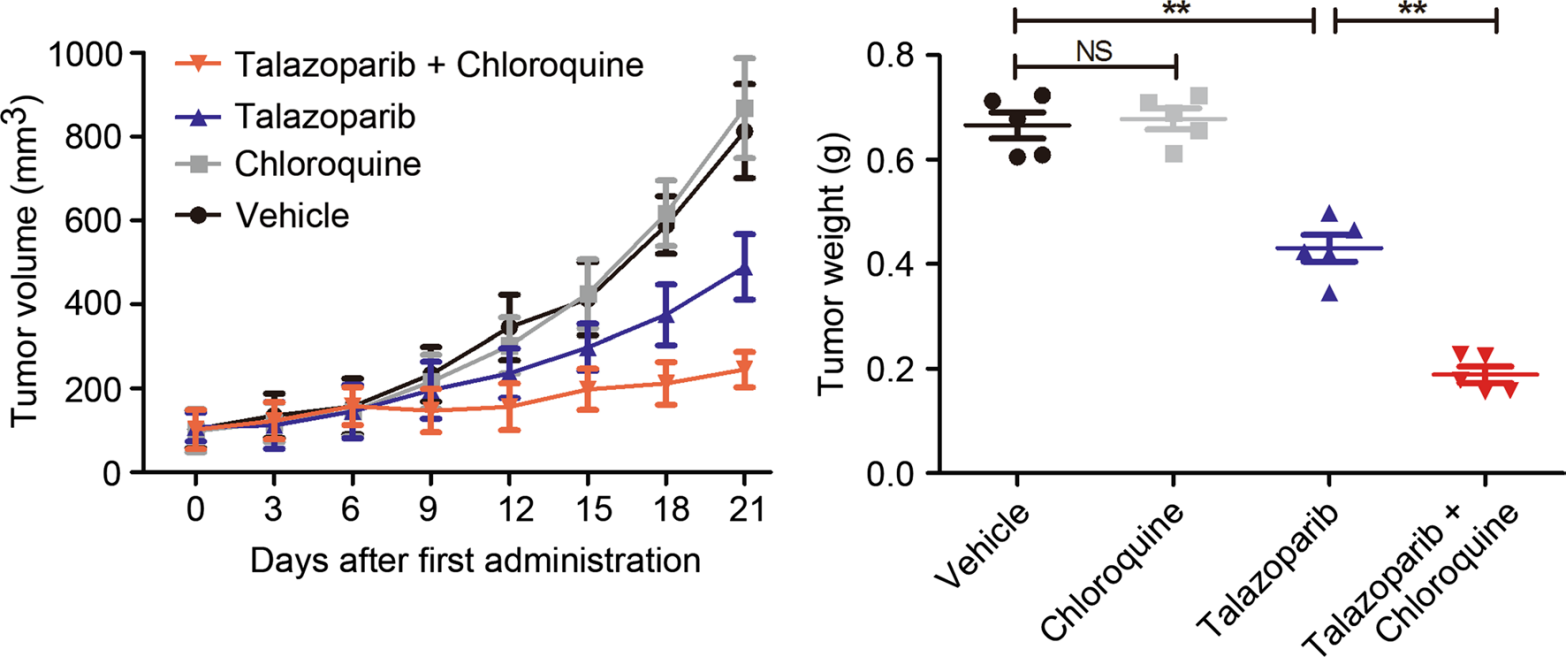
Targeting PARP and autophagy elicited synergetic anti-tumor effect in pediatric CML. Patient-derived xenograft (PDX) model was established to evaluate the anti-tumor efficacy of talazoparib and/or chloroquine in CML. Tumor volume was measured once every 3 days. After treatment with talazoparib and/or chloroquine for 21 days, tumor weight was shown as mean ± SD. Each point indicated an independent value from one mouse (n = 5). *NS* no significance; **P < 0.01

To confirm the therapeutic effect of talazoparib in combination with autophagy inhibitor for pediatric CML treatment, PDX model was established. In Fig. 7, tumor volume showed no remarkable difference among vehicle control group and chloroquine groups. However, tumor volume reduced significantly from day 6 in talazoparib and chloroquine co-treated group (*P* < 0.01). After treatment with talazoparib and/or chloroquine for 21 days, mean tumor weight of vehicle, chloroquine, talazoparib, talazoparib in combination with chloroquine were 665.4 ± 55.85 mg, 678 ± 44.75 mg, 430 ± 57.56 mg, 188.8 ± 35.06 mg. These data showed that talazoparib could inhibit CML growth in vivo. When CML-bearing mice were administrated with talazoparib and chloroquine, the anti-tumor effect was significantly increased (Fig. 7). Thus, talazoparib showed potent anti-CML effect in vivo, inhibition of autophagy by chloroquine potentiated the anti-CML effect of talazoparib.

## Discussion

To date, PARP inhibitors such as olaparib, rucaparib, niraparib and talazoparib have been approved by FDA to treat BRCA-mutated ovarian and breast cancers (Franzese et al. 2019). Clinical application of those PARP inhibitors offered beneficial effects to patients by its unique ability to selectively kill the cancer cells with homologous recombination deficiency (Karantza-Wadsworth et al. 2007). Talazoparib is an oral PARP inhibitor and was newly approved in the USA for the treatment of locally advanced and metastatic HER2-negative breast cancer. Talazoparib also underwent the development for use in metastatic castration-resistant prostate cancer and early triple negative breast cancer (Hoy 2018). However, whether PARP inhibitor talazoparib could elicit cytotoxicity in CML cells was still unclear. In current study, it was the first report to evaluate the anti-tumor effect of talazoparib in CML. Our results showed that talazoparib treatment induced a concentration-dependent cytotoxicity in primary CML cells and reduced the tumor growth in PDX model.

Although accumulating evidences show that PARP inhibitor has potent anti-tumor effect in some malignancies, strategies to enhance the effect are still desperately needed (Scott et al. 2015). To further enhance the anti-tumor effect of talazoparib in CML, we examined the status of autophagy in talazoparib-treated CML cells. Although autophagy showed as a “double-edged sword” in carcinogenesis, increasing literatures indicated the cyto-protective function of autophagy in tumor treatment and autophagy has been regarded as a potential target for synergetic anti-tumor therapeutics (He and Klionsky 2009). Literatures showed that targeting autophagy-related proteins LC3, ATG5, ATG7, SQSTM1, and Akt/mTOR (mammalian target of rapamycin) pathway could regulate autophagy initiation in cancer cells (Wen et al. 2018). In the current study, we reported that autophagy was markedly triggered by talazoparib in CML cells, which was confirmed by the accumulation of autophagosomes, decrease of SQSTM1 and up-regulation of LC3-II.

Assessment of the function of autophagy in PARP inhibitor-based tumor treatment was still rare. To address this point, pharmaceutical inhibitor and siRNA were employed to block talazoparib-induced autophagy in CML cells. Though autophagy played a crucial role in both cell death and cell survival (Washington et al. 2015), our results showed that inhibition of autophagy significantly increased talazoparib-triggered cytotoxicity in CML cells, indicating autophagy as a cyto-protective mechanism in talazoparib treatment in vitro. In CML PDX model, we investigated the anti-tumor effect of talazoparib in combination with chloroquine and found that autophagy inhibitor chloroquine significantly potentiated the anti-tumor effect of talazoparib. Taken together, our results demonstrated that PARP could be a promising target for CML treatment and highlighted the synergetic anti-tumor effects of co-targeting PARP and autophagy, providing novel insights for CML treatment.

## Data Availability

Please contact the author for data request.

## Abbreviations

PARP: poly (ADP-ribose) polymerases
CML: chronic myeloid leukemia
siRNA: small-interfering RNA
SCR: non-silencing scrambled control
PDX: patient-derived xenograft
SQSTM1: sequestosome 1
LC3: microtubule-associated protein 1 light chain3
mTOR: mammalian target of rapamycin
